# Impaired microcirculation after subarachnoid hemorrhage in an *in vivo* animal model

**DOI:** 10.1038/s41598-018-31709-7

**Published:** 2018-09-06

**Authors:** Kuo-Chuan Wang, Sung-Chun Tang, Jing-Er Lee, Jui-Chang Tsai, Dar-Ming Lai, Wei-Chou Lin, Chih-Peng Lin, Yong-Kwang Tu, Sung-Tsang Hsieh

**Affiliations:** 1 Division of Neurosurgery, Department of Surgery, National Taiwan University Hospital, Taipei, Taiwan; 2 Department of Neurology, National Taiwan University Hospital, Taipei, Taiwan; 3 Department of Neurology, Taipei Medical University-Wan Fang Hospital, Taipei, Taiwan; 4 Department of Anatomy and Cell Biology, College of Medicine, National Taiwan University, Taipei, Taiwan; 5 Department of Pathology, National Taiwan University Hospital, Taipei, Taiwan; 6 Department of Anesthesiology, National Taiwan University Hospital, Taipei, Taiwan

## Abstract

The influence of aneurysmal subarachnoid hemorrhage (SAH) on brain microcirculation has not yet been systematically investigated. We established an animal model to examine (1) the brain surface microcirculation (2) the influences of cerebrospinal fluid (CSF) from aneurysmal SAH on the brain surface microcirculation. A rat SAH model was induced by injection of autologous arterial blood into the cisterna magnum, and the brain surface microcirculation was evaluated by a capillary videoscope with craniotomy at the fronto-parietal region. CSF from SAH rats and SAH patients was applied on the brain surface of naïve rats to assess the resulting microcirculatory changes. In the SAH rats, diffuse constriction of cortical arterioles within 24 hours of SAH was observed. Similar patterns of microcirculation impairment were induced on normal rat brain surfaces via application of CSF from SAH rats and SAH patients. Furthermore, the proportion of subjects with arteriolar vasoconstriction was significantly higher in the group of SAH patients with delayed ischemic neurological deficits (DIND) than in those without DIND (*p* < 0.001). This study demonstrated impaired microcirculation on brain surface arterioles in a rat model of SAH. CSF from SAH rats and patients was responsible for impairment of brain surface microcirculation.

## Introduction

Conventional wisdom holds that vasospasm of major cerebral vessels following aneurysmal subarachnoid hemorrhage (SAH) is responsible for detrimental neurological outcomes. Despite this prevailing hypothesis, a growing number of reports have suggested the existence of alternative mechanisms of neurological deficits^1,2^; for example, ischemic brain injury could develop within the normal range of cerebral perfusion pressure^3,4^. Furthermore, pathological examination of dogs after experimental SAH showed luminar narrowing of the intraparenchymal small arteries or arterioles on the brain surface^5^. These findings raised a critical issue, i.e., whether or not microcirculation is altered after aneurysmal SAH.

The development of models of SAH is essential in order to address the issue of microcirculation impairment after SAH and to explore the mechanisms and mediators underlying SAH-induced injury. Previously-developed models include cisternal injection of autologous blood and filament perforation^6,7^. Limited studies have investigated the pathology of microvasculatures on the cortical surface responsible for microcirculation^1^. In addition to initial bleeding insults, several factors including hydrocephalus, cerebral edema, and delayed ischemic neurological deficits (DIND) contribute to secondary brain injury^8–11^. Among these factors, DIND has been proposed to be one of the major factors responsible for a poor outcome in the postoperative period^12,13^. The underlying pathophysiology of DIND, however, remains obscure. Most studies have suggested associations of DIND with angiographic vasospasm^14–20^; however, the correlation between the frequency of DIND and angiographic vasospasm is controversial^19^. For example, some patients have not developed DIND, despite objective evidence of severe vasospasm on cerebral angiograms or transcranial Doppler examinations^21–23^.

In this study, we aimed to examine the effect of SAH on brain surface microcirculation in a rat model of SAH, and assessed: (1) pathological alterations of microcirculation vasculature, and (2) physiological changes in cerebral regional blood flow and tissue oxygenation. We further extended this experimental system in order to assess the effect of CSF from SAH patients on brain surface microcirculation and its relationship with DIND after aneurysmal SAH.

## Materials and Methods

This study included animal experiments and experiments on human subjects, which were approved by the Institutional Review Board of National Taiwan University Hospital, Taipei, Taiwan, and the Animal Committee of National Taiwan University College of Medicine. All experiments and protocols were conducted in compliance with human and animal ethical regulations.

### Animal preparation

Male Wistar rats, weighing 250~300 g, were anesthetized with 1.5% halothane in a mixture of 30% O_2_/70% N_2_O. The right femoral artery was cannulated for continuous arterial pressure monitoring. Systemic arterial pressure was monitored using an RFT Biomonitor, VEB (Messgeraetewerk, Zwoenitz, Germany). Body temperature was maintained at 37 ± 0.5 °C by using a heating pad. The depth of anesthesia was monitored by response to tail pinching.

### Rat SAH model

An SAH model was induced in rats following a standard protocol by injecting 0.3 ml of fresh autologous blood into the cisterna magna over a 5-min period with the animal in a 20-degree head-down position^24^. After the procedure, the rat was placed in the prone position with gauze compression for 5 min to stop reflux of blood from the needle hole after injection.

### Craniotomy for microcirculation observation

**Figure 1:**
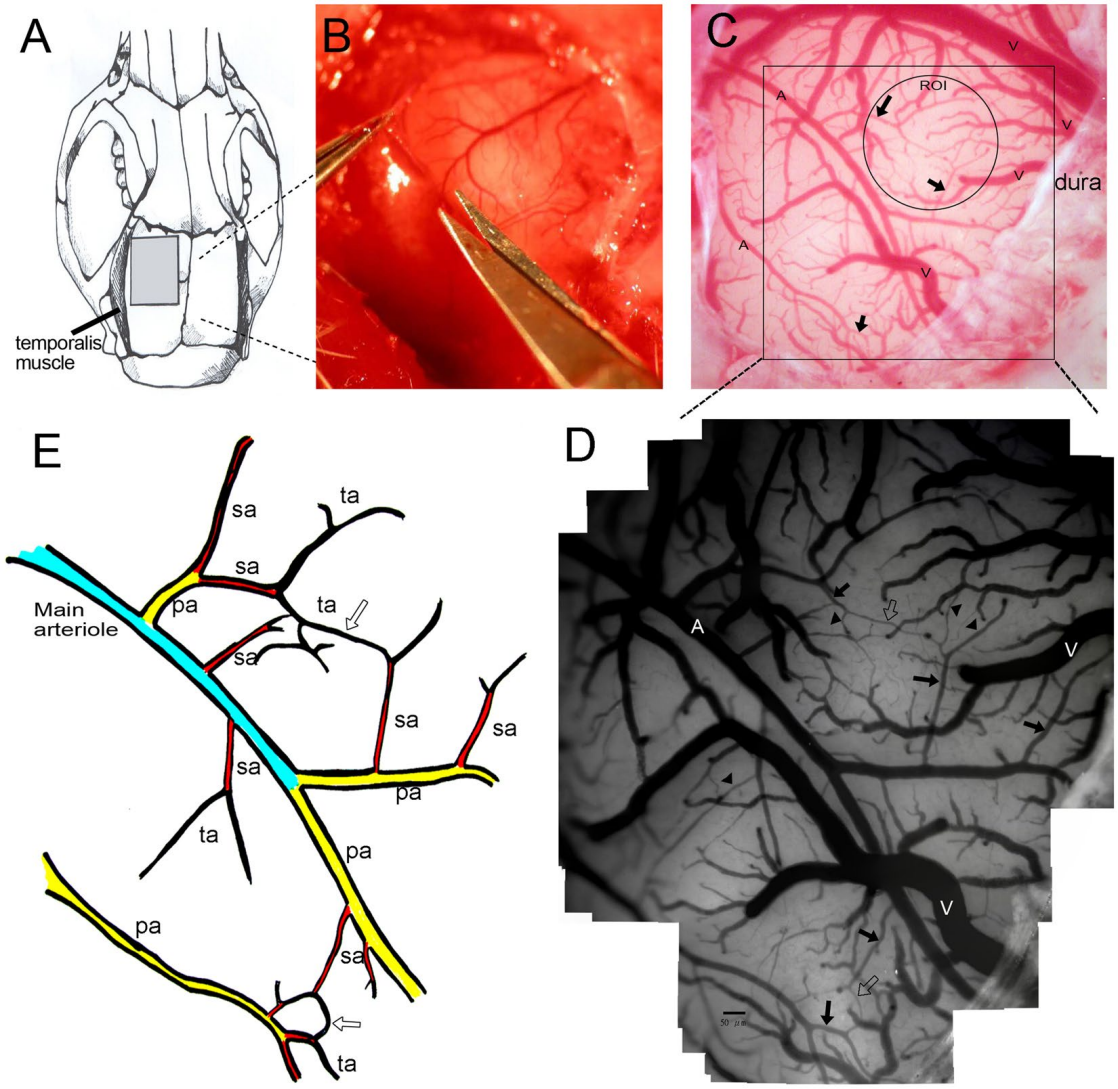
The microcirculation system on the cortical surface of the rat brain. (**A**) A craniotomy of 5 × 5 mm^2^ was made behind the frontal suture. (**B**) The dura was opened using micro-scissors. (**C**) Under a dissection microscope equipped with a capillary videoscope at a resolution of 0.91 μm/pixel, all vasculatures on the brain surface, including the main arterioles and venules, were clearly seen (marked A and V to represent arterioles and venules, respectively). (**D**) This figure was created as a montage of 25 individual photos in order to obtain a panoramic view. In each craniotomy field, there were 1–2 main arterioles, which branched into primary arterioles (pa), secondary arterioles (sa), and terminal arterioles (ta). Terminal arterioles formed collateral circulation systems (hollow arrow) with each other, and finally disappeared from the brain surface. (**E**) Schematic diagram showing the microcirculation system on the brain surface.

A craniotomy measuring 5 × 5 mm^2^ was performed at the left frontal suture using a saline-cooled drill. The dura mater was removed and hemostasis was achieved by packing with gauze (Fig. 1A,B).

### Examination of microcirculation on the brain surface of rats

Brain surface microcirculation was examined using a capillary videomicroscope, a CAM1 capillary Anemometer (KK Technology, London, United Kingdom), which consisted of a monochrome charge-coupled device (CCD) video camera with a resolution of 752 × 582 pixels. A dissection microscope was attached to allow 3-dimensional adaptations without contact with the brain surface. The field of vision was 684 × 437 μm^2^, and the image was enlarged to give an overall magnification of 0.91 μm/pixel.

### Patterns of brain surface microcirculation

According to previous literature^25^, cerebral meninges are rich in blood vessels but there are no capillaries^25,26^. By human pathology study, the arteries on the cortical surface were classified into four parts: the conducting artery, distributing artery, precortical artery, and cortical artery^26^. In the rat, we observed replication of this pattern of distribution by capillary videoscope (Fig. 1D,E). There were 1~2 main arterioles in each field of the craniomy site. Each main arteriole was divided into 2~4 primary arterioles (pa), followed by secondary arterioles (sa), and finally terminal arterioles (ta) (ta was defined as the terminal arterioles which could be seen on the brain surface, and sa is the arterioles which ta branched from.). There existed extensive collateral circulation between ta. According to study of the human cortex by scanning electron micrograph, ta penetrate into the cortex at right angles and supply the capillary networks beneath the cortex^26^.

### Quantification of diameters of the pa, sa, and ta in the SAH model

To quantify the microcirculation on the rat cortical surface, we reconstructed the entire field of the craniotomy via a montage approach, integrating all photographs in that field (Fig. 1) using Photoshop software (Version CS6; Adobe Systems, San Jose, CA, USA). First, we identified the main arterioles (Fig. 1E), then took photographs along the arterioles and all the branches until communication with the territory of another main arteriole. Each field used for study covered approximately 3 × 3 mm^2^. For each field, in order to be complete, >10 photographs were required (15.1 ± 3.2 photographs per field). We then sampled all vessels in that territory and identified them as main arterioles, pa, sa, and ta, and then measured the diameters of the sampled arterioles individually. The abundance of arterioles in each frame of 3 × 3 mm was similar between the control and SAH rats, including the sa (7.1 ± 2.2 vs. 6.9 ± 2.0/mm^2^, *p* = 0.996) and ta (19.9 ± 5.3 vs. 19.1 ± 3.4/mm^2^, *p* = 0.887).

### Measurement of brain regional blood flow and partial pressure of oxygen in brain tissue (PbtO_2_)

The extent of tissue perfusion and PbtO_2_ in the brain cortex were measured using an OxyLite 2000E detector and an OxyFLO 2000E detector (Oxford Optronic Ltd, England)^27^. The probe was fixed on a stereotactic frame (Stoelting, Wood Dale, IL, USA), and the tip was inserted to a depth of 2 mm underneath the brain surface to start recording. The tip was then placed 1 mm deeper for each subsequent recording until the probe tip was 7-mm-deep in the cerebral cortex. Continuous recording of brain perfusion and blood pressure was performed during the whole course.

### SAH patients and management protocol

The study recruited 20 to 80 year-old patients with Modified Fisher’s Grade III/IV aneurysmal SAH. Patients with intracerebral hemorrhage resulting in midline shift or intraventricular hemorrhage causing obstructive hydrocephalus were excluded. Written informed consent was obtained from each patient or from a legal representative of patients with decreased consciousness.

To investigate the characteristics of the CSF after SAH, a lumbar drainage tube was inserted on the 3rd day after SAH, and continuous drainage was maintained at a rate of 20–80 ml per 8 hours in order to maintain an intracranial pressure of 5–15 mmHg. This procedure was approved by the Institutional Review Board of National Taiwan University Hospital, and consent forms were obtained prior to commencement of lumbar drainage. All patients received standard medical management for vasospasm with nimodipine, hypervolemia, and induced hypertension.

### Definition of DIND

DIND was defined according to the conventional consensus^28^ as clinical deterioration or a new infarct on a CT scan that was not visible upon admission or on immediate postoperative scans. Other causes of clinical deterioration or CT hypo-densities, including hydrocephalus, re-bleeding, cerebral edema, retraction injury, meningitis, and seizures, were excluded. The diagnosis of DIND was adjudicated by the entire research team.

### CSF sample collection, preparation, and analysis

CSF samples from SAH rats and patients were obtained from the cisterna magna (rats) and the lumbar drainage tube (patients). In the SAH patients, CSF from the intrathecal space was drawn on the first day after lumbar drainage. The CSF was centrifuged at 3000 ppm for 10 min, and the supernatant was maintained at 30 °C for immediate animal studies. CSF samples from non-SAH control patients were used as the control group. CSF was collected from patients undergoing removal of an internal fixation wire during spinal anesthesia.

### Experimental paradigms of the effects of rat and human CSF on microcirculation

Digital images of the brain microcirculation were recorded using a CAM1 microscope continuously for 2 hours after CSF superfusion. Room temperature was maintained at 28 °C. Normal saline and CSF were maintained in a water bath at 30 °C. Topical brain superfusion was performed using normal saline for 10 min after opening the dura. When a stable condition was attained, gauze was used to suck the saline from the dural edge without touching the brain surface, and then CSF drawn from SAH rats and patients was superfused respectively on the cortical surface. Images were taken at 1, 5, 30 and 120 min for analysis. Artificial CSF (Artcereb, Otsuka Pharmaceutical Factory, Japan) and CSF from non-SAH patients were used as controls.

### Measurement of microcirculation changes following superfusion of CSF from SAH rats and patients

To test the effects of CSF from SAH rats and patients on microcirculation, we applied CSF to the brain surface of a naïve rat after craniotomy, as described in the preceding section concerning the rat SAH model. The quantitative method used to assess the diameters of the pa, sa, and ta was as described above. CSF was superfused on the surface of the brain cortex, ensuring full coverage of the brain surface under the craniotomy of an area of 5 × 5 mm^2^. The diameter of each vessel was measured again 120 min after the superfusion of CSF. The percentage change in diameter of each vessel before vs. after CSF superfusion was calculated according to the formula: change in diameter before – diameter after CSF superfusion/diameter before CSF superfusion × 100%. The mean change of all sa in the field under CSF superfusion was designated as the % of vessel constriction in the sa for that case (i.e., in each individual rat or patient). The % of vessel constriction in the ta was calculated according to the same definition.

### Brain preparation for immunohistochemical analysis

Rats were sacrificed by injection of pentobarbital (200 mg/Kg, i.p.) and perfused transcardially with 50 ml of saline followed by 500 ml of a fixative containing 4% paraformaldehyde in 0.1 M phosphate-buffered saline (PBS) at pH 7.3 for 30 min. Immunohistochemical analysis was performed on cryostat sections or paraffin-embedded sections according to the requirement of each individual antibody: (1) serial coronal brain sections of a thickness of 6 µm were cut on a cryostat and mounted onto gelatin-coated slides for immunohistochemical staining, and (2) sections were de-paraffinized by heating at 60 °C for 30 min and xylene treatment, rehydrated by passing through a series of decreasing concentrations of ethanol (100%, 90%, 70%, and 50%) for 5 min for each step, and then washed with 0.1 M PBS.

Endogenous peroxidase was quenched with 3% hydrogen peroxidase for 10 min. The sections were incubated with 5% bovine serum albumin for 1 hour to block non-specific background staining, then subsequently incubated with each primary antibody overnight at 4 °C and visualized using a Novolink Polymer Detection System (RE7140-K; Novocastra, Newcastle upon Tyne, UK).

Antibodies against the following molecules were used in this study: fibrin (A0080, DakoCytomation, Glostrup, Denmark), glucose transporter 1 (GLUT1) (ab652, Abcam, Cambridge, United Kingdom), and α-smooth muscle actin (α-SMA) (A5228; Sigma, St. Louis, MO, USA).

### Quantitation of thrombosed vessels

Double immunofluorescence staining for GLUT1 and fibrin was performed for morphological analysis of vasculatures (thrombosed vs. patent vessels). Rat brain sections were incubated with primary antibodies against GLUT1 and fibrin at 4 °C overnight, and a secondary antibody was then applied (Alexa Fluor® 594, ThermoFisher, A21203) at room temperature for 30 min. Each immunofluorescence-stained section was digitized for measurement of the area of GLUT1 (+) vessels and the area of fibrin (+) thrombosed vessels using a 3-CCD color video camera (Zeiss Axiovert 200 M, Germany) interfaced with the MetaMorph image analysis system. The proportion of thrombosed vessels was calculated according to the following formula: fibrin (+)/GLUT1 (+) vessel area divided by GLUT 1 (+) vessel area.

### Statistical analysis

Statistical analyses were performed using SAS software, Version 9.1.3 (SAS Institute Inc., Cary, NC, USA). All values were expressed as means and standard errors of the mean. Comparisons between groups were made using t-tests, chi-square tests, or Fisher’s exact tests according to the distribution or characteristics of the data. The goal of regression analysis was to identify one or several *parsimonious* regression models that fitted the observed data well for outcome prediction or effect estimation. To ensure the quality of the analyses, basic model-fitting techniques for (1) variable selection, (2) goodness-of-fit (GOF) assessment, and (3) regression diagnostics were used in our regression analyses. Specifically, a stepwise variable selection procedure was applied to obtain the candidate final regression model. *p* < 0.05 was considered statistically significant.

## Results

### Impairment of brain surface microcirculation in a rat SAH model

**Figure 2:**
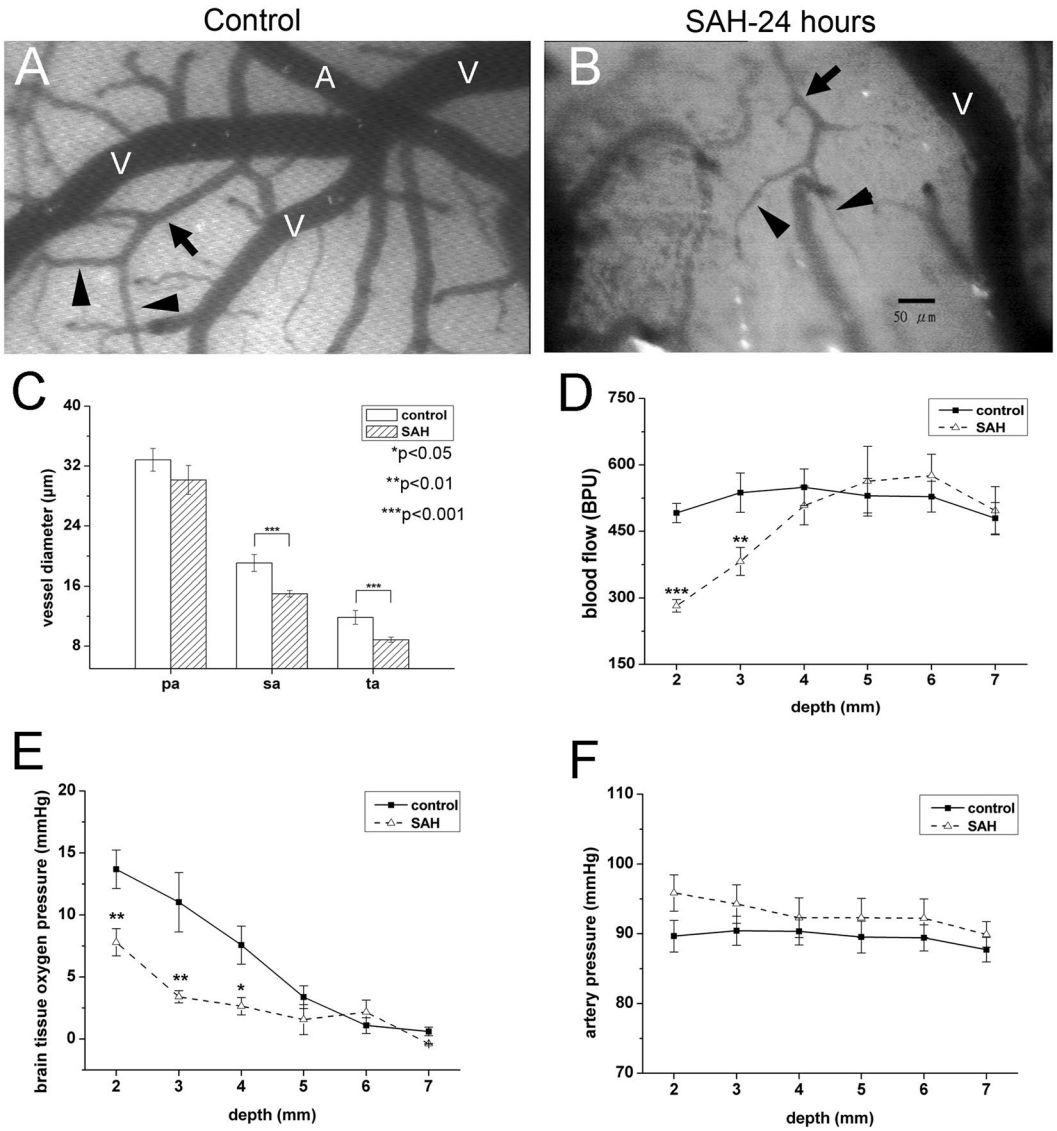
Changes in microcirculation after subarachnoid hemorrhage (SAH). The microcirculation on the brain surface of rats after craniotomy was examined 24 hours after experimental SAH, as detailed in Materials and Methods. Microcirculation parameters included arterial diameter (**C**) regional cerebral blood flow (**D**) partial pressure of oxygen in brain tissue (PbtO_2_) (**E**) and arterial pressure (**F**). (**A**,**B**) Diffuse vasoconstriction of secondary arterioles (sa, arrows) and terminal arterioles (ta, arrowheads) was observed in the SAH group (SAH, **B**) as compared with the control group (control, **A**). (**C**) Quantitatively, the diameters of the sa and ta were smaller in the SAH group than in the control group. The size of the primary arterioles (pa) was similar in both groups. (**D**,**E**) The regional cerebral blood flow (**D**) and PbtO_2_ (**D**) at the brain surface were significantly lower in the SAH rats than in the control rats at a depth of <4 mm from the brain surface. (**E**) The arterial pressure at the brain surface (at a depth of <3 mm) was higher in the SAH group than in the control group. *p < 0.05, **p < 0.01.

**Figure 3:**
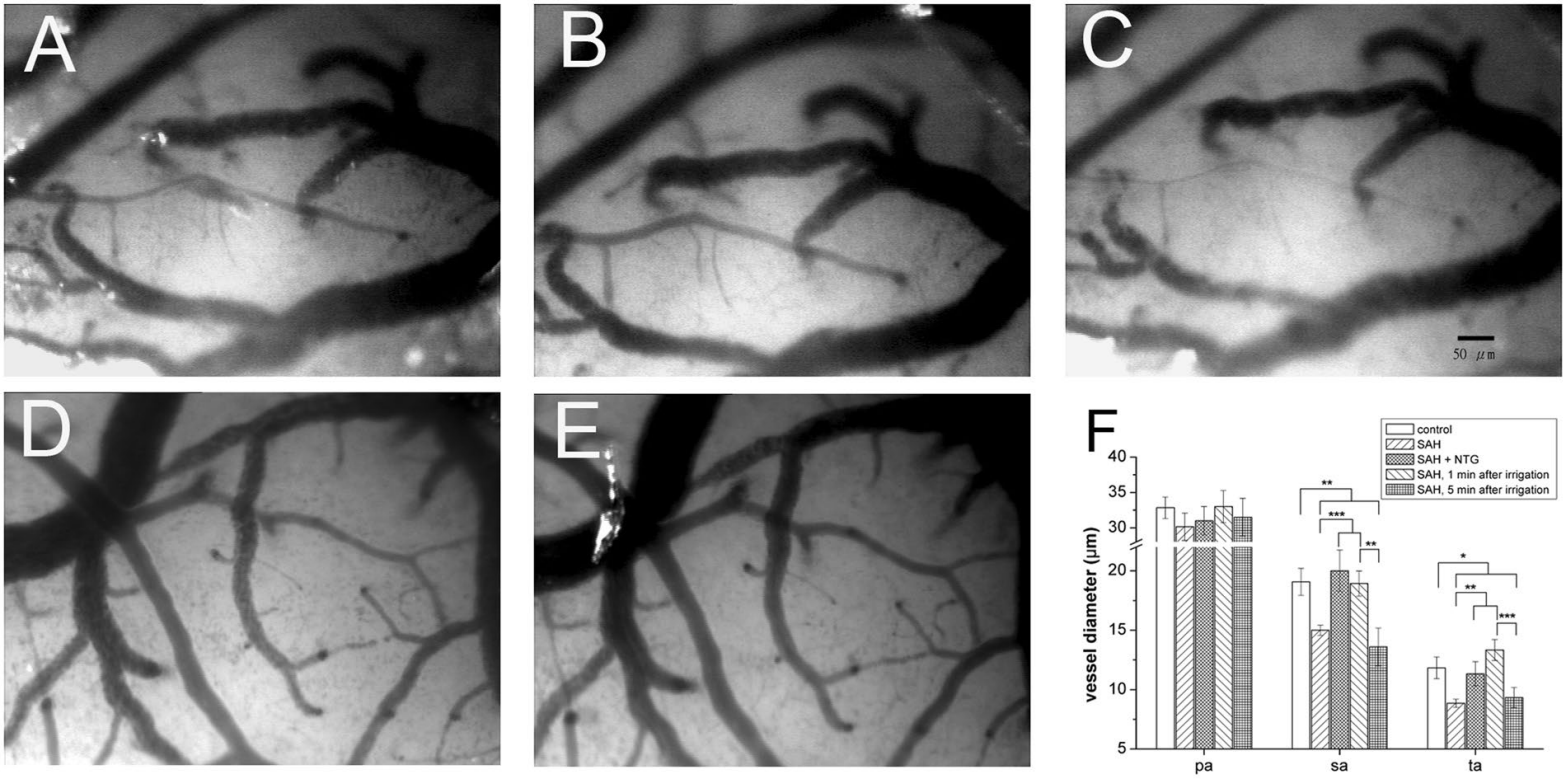
Vaosconstriction of small arterioles after subarachnoid hemorrhage (SAH) was reversible. The microcirculation on the brain surface of rats after craniotomy was examined 24 hours after experimental SAH, as detailed in Materials and Methods. (**A**,**D**) Marked vasoconstriction was observed in arteriole 24 hours after SAH. (**B**) The vasoconstrictive effect was alleviated by normal saline irrigation initially, but rebound vasoconstriction noted 5 min after irrigation (**C**). (**E**) The vasoconstrictive effect was reversible by application of nitroglycerin (NTG). (**F**) Quantitatively, the diameters of the sa and ta were smaller in the SAH group than in the control group. The size of the primary arterioles (pa) was similar in both groups. The vasoconstrictive effect could be alleviated by normal saline irrigation or NTG application. *p < 0.05, **p < 0.01, ***p < 0.001.

To understand the alterations in cortical surface microcirculation, we first established an animal model of SAH by injecting autologous blood into the cistern magnum of rats. The abundance of arterioles in each frame of 3 × 3 mm was similar in the control and SAH rats, including the sa (7.10 ± 2.15 vs. 6.90 ± 2.01 arterioles/mm^2^ *p* = 0.99) and ta (19.9 ± 5.34 vs. 19.1 ± 3.35 arterioles/mm^2^, *p* = 0.89). Within 24 hours after SAH, the diameters of the arterioles on the cortical surface were markedly reduced in the sa (15.00 ± 0.48 vs. 19.10 ± 1.14 μm, *p* < 0.01) and ta (8.70 ± 0.42 vs. 11.80 ± 0.92 μm, *p* < 0.01) (Fig. 2B,C). The vasoconstriction on the brain surface of the SAH rats could be reversed after irrigation with normal saline or nitroglycerin (Fig. 3).

In our SAH model, there were decreased tissue perfusion and oxygenation, especially in the superficial brain region. As shown in the Fig. 2D,E, the regional tissue perfusion as assessed by cerebral blood flow and the tissue oxygenation by measurement of brain tissue oxygen partial pressure (PbtO_2_) were significantly lower in SAH vs. control (all *p* < 0.05 at depths of 2 mm, 3 mm and 4 mm; SAH vs. control). During the measurements, arterial PaO2 showed no difference between SAH and control group (SAH vs. control, 79.2 ± 4.45 vs. 82.4 ± 5.00, *p* = 0.308), but blood pressure was slight elevated in SAH group (*p* = 0.094) (Fig. 2F).

The severity of impairment of tissue perfusion and the degree of reduction of PbtO_2_ were related to the distance of the cerebral cortex from the surface; i.e., the more superficial the layer, the lower the tissue perfusion. In the cortex at levels deeper than 5 mm from the cortical surface, there was no change in tissue perfusion (SAH vs. control, *p* = 0.75, *p* = 0.38, *p* = 0.65 at a depth of 5 mm, 6 mm, 7 mm, respectively) or PbtO_2_ (SAH vs. control, *p* = 0.54, *p* = 0.68, *p* = 0.71 at a depth of 5 mm, 6 mm, 7 mm, respectively).

The constricted arterioles dilated immediately after normal saline irrigation [diameters 1 min before and after irrigation: sa (15.0 ± 0.48 vs. 18.9 ± 1.06 μm, *p* = 0.001) and ta (8.7 ± 0.42 vs. 13.7 ± 0.89 μm, *p* = 0.001)]. However, these vessels became constricted again within 5 min after the end of irrigation [diameter 1 min before vs. 5 min after irrigation: sa (15.0 ± 0.48 vs. 13.6 ± 1.59 μm, *p* = 0.414) and ta (8.7 ± 0.42 vs. 9.3 ± 0.84 μm, *p* = 0.541)], indicating the persistence of vasoconstricting factors in the SAH rats (Fig. 3A–C,F). Reversibility of SAH-induced vasoconstriction was also documented following nitroglycerin application: sa (15.0 ± 0.48 vs. 19.1 ± 1.14 μm, *p* = 0.001) and ta (8.7 ± 0.42 vs. 11.8 ± 0.92 μm, *p* = 0.007) (Fig. 3D–F).

### Pathology of impaired microcirculation in the SAH model: vasoconstriction of cortical arterioles and diffuse thrombosis of capillaries

**Figure 4:**
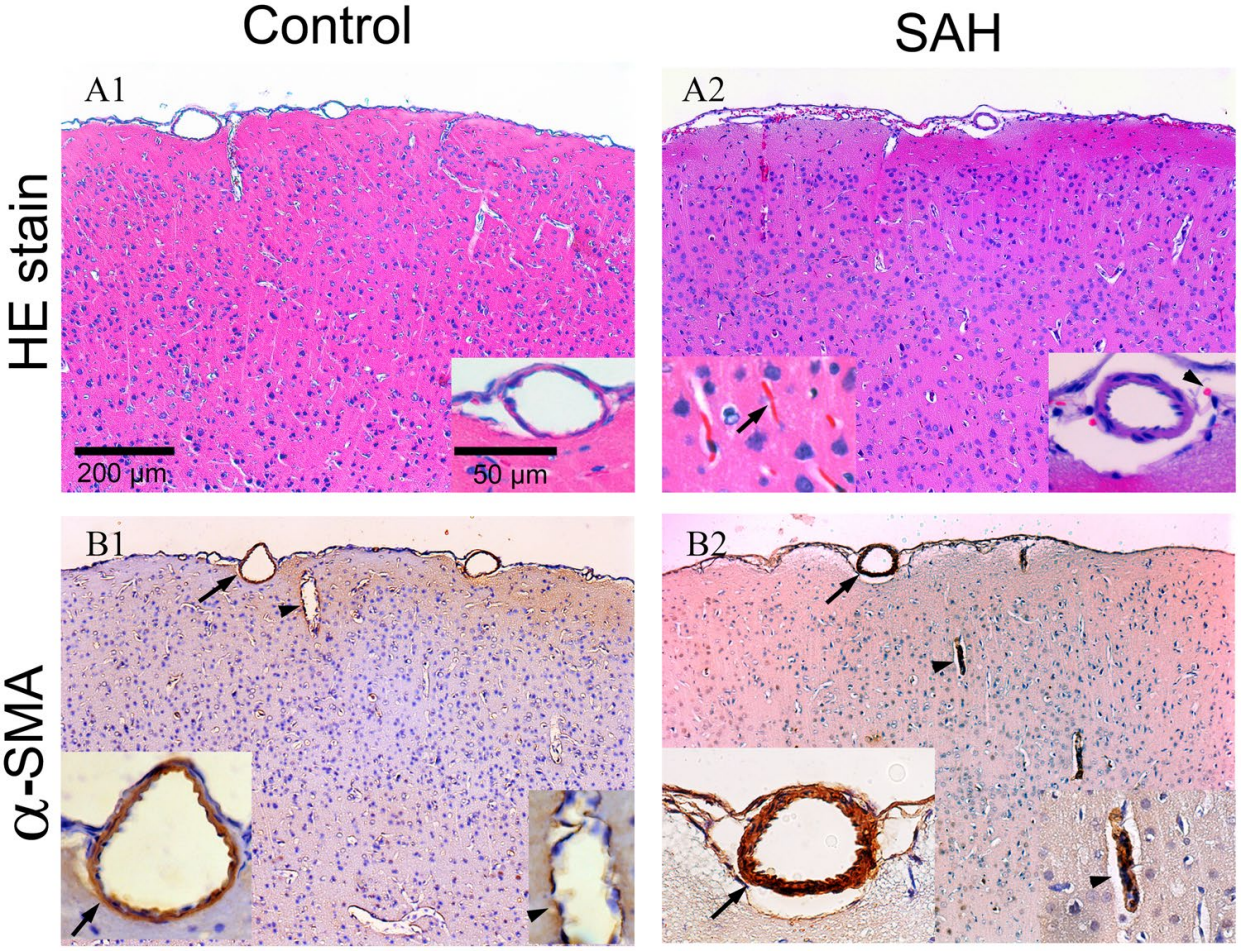
Pathological examination of rat brains 24 hours after SAH. Rat brains were dissected and processed for paraffin-embedding. Sections were then stained with H&E and subjected to immunohistochemical analysis. (**A**) Red blood cells were seen in the subarachnoid space (arrowhead) in the rat SAH model. Diffuse thrombosis of small vessels beneath the cortex (black arrow) was demonstrated by H&E staining. (**B**) Vasoconstriction of the cortical vessel (black arrow) and perpendicular branches (arrow head) of the vessels was demonstrated by staining with α-smooth muscle actin (α-SMA).

To investigate the pathological substrates of impaired brain surface microcirculation, we examined the changes in brain vasculatures. Diffuse thrombosis of capillaries in the cerebral cortex was observed (Fig. 3), and vasoconstriction of the cortical vessel and perpendicular branches was demonstrated by α-smooth muscle actin (α-SMA) staining (Fig. 4).

**Figure 5:**
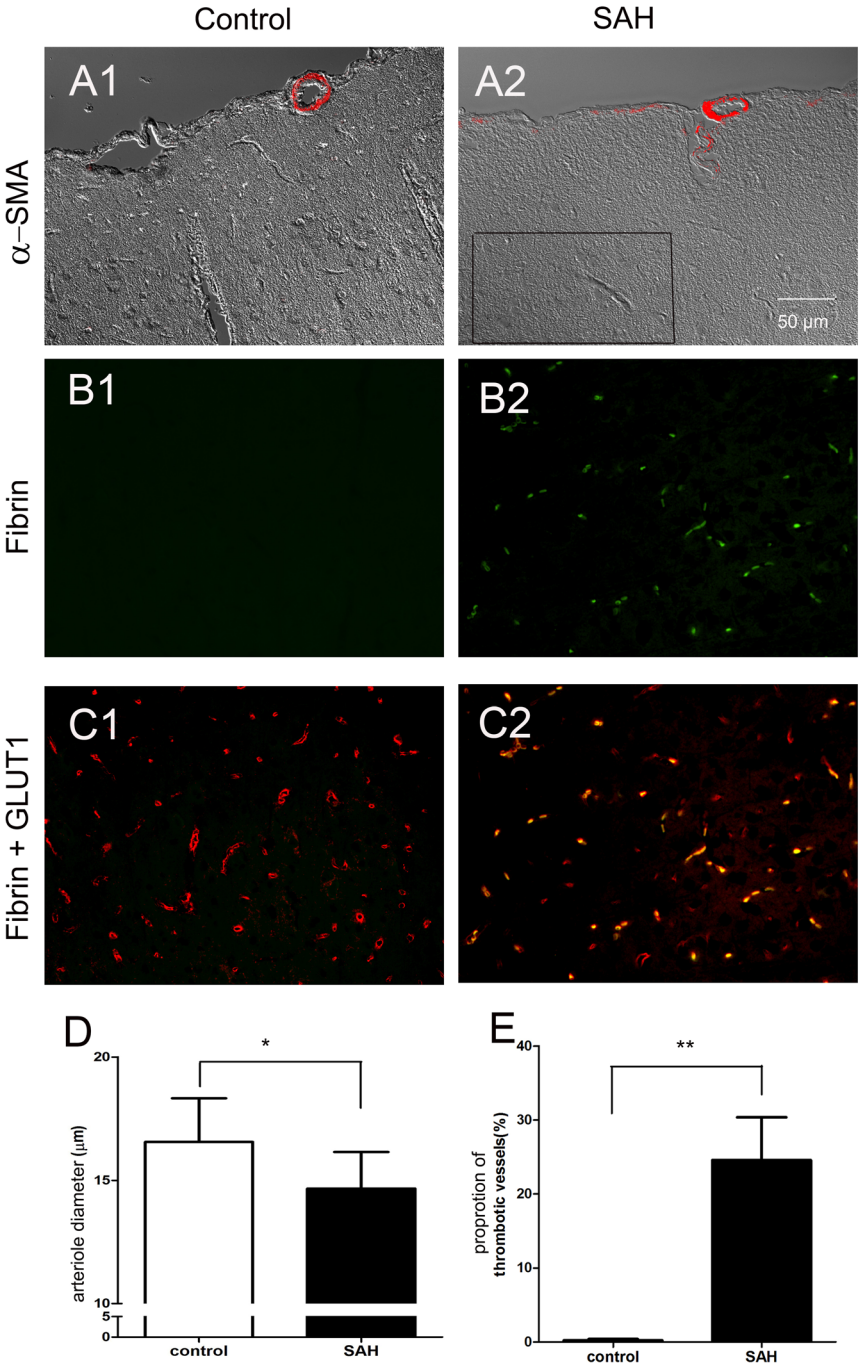
Vasoconstriction and thrombosis in the rat brain 24 hours after subarachnoid hemorrhage (SAH). Rat brain tissues were immunofluorescently stained with α-smooth muscle actin (α-SMA) for arterioles. Capillaries and thrombi inside capillaries were labeled with glucose transporter 1 (GLUT1, red) and fibrin (green), respectively. (**A**) Cortical arterioles were constricted in the SAH group as compared with the control group. (**B**) Fibrin (+) thrombi were detected in the capillaries of the brain parenchyma in the SAH group, but were absent in the control group. (**C**) The proportion of thrombosed capillaries (yellow) was demonstrated by double-labeling immunofluorescence of fibrin (green) and GLUT1 (red). (**D**,**E**) The arteriolar diameter and the proportion of fibrin (+) capillaries were quantitatively analyzed. Bar = 50 μm.

To further characterize the structural alteration of cortical arterioles, we quantified (1) the diameters of cortical arterioles, and (2) the proportion of thrombosed capillaries using double-labeling immunofluorescence of fibrin and glucose-transporter 1 (GLUT1), a marker of the endothelium (Fig. 5). The arteriolar diameter 24 hours after SAH was significantly smaller than that of the control group (14.67 ± 1.45 μm vs. 16.55 ± 1.69 μm, *p* = 0.007) (Fig. 5D). The proportion of thrombosed capillaries 24 hours after SAH was significantly higher than that of the control group (24.6% ± 5.8% vs. 0.5% ± 0.4%, *p* = 0.01) (Fig. 5E).

### Superfusion of CSF from SAH rats on the brain surface of control rats induced rapid microcirculatory changes

**Figure 6:**
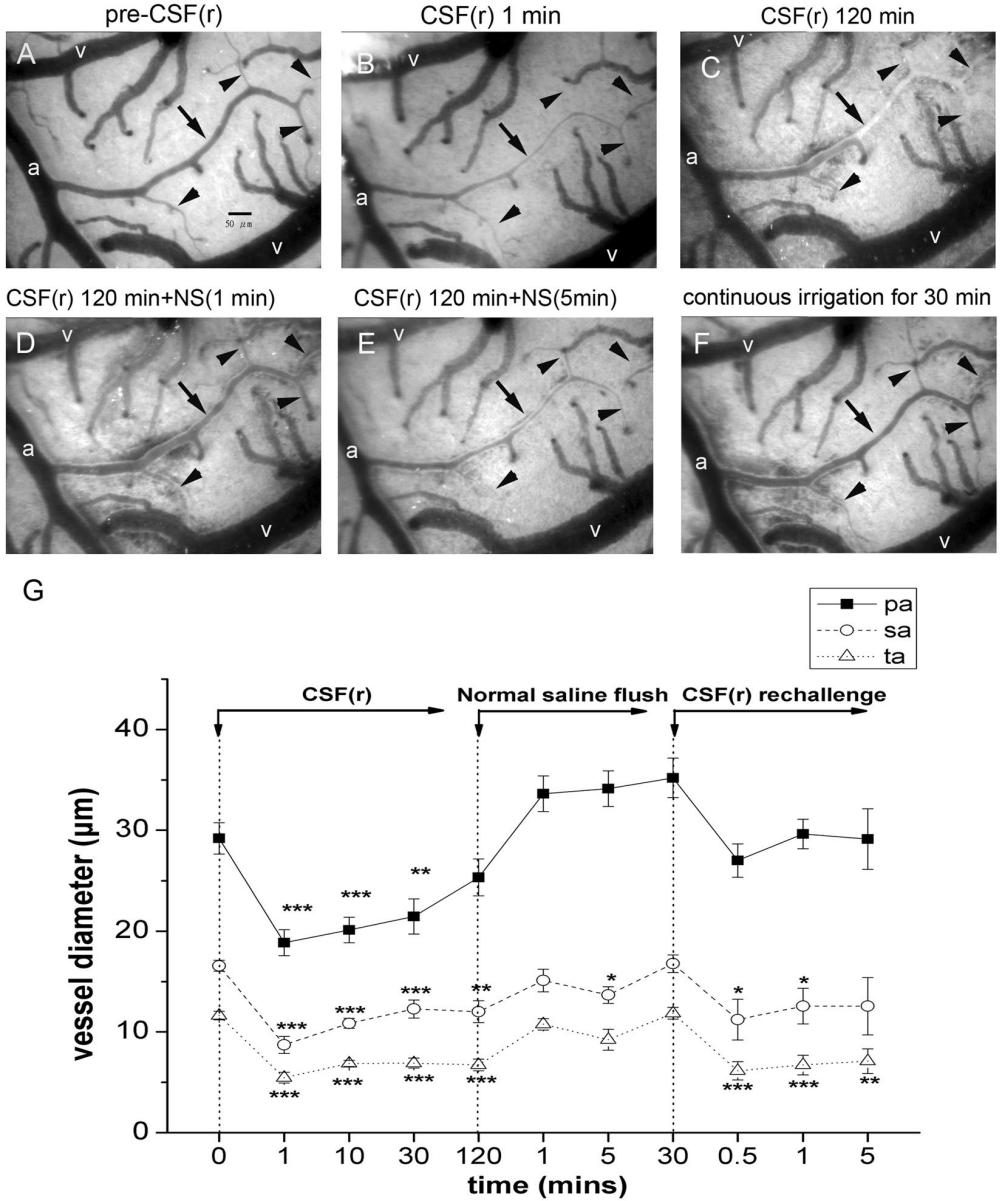
Effects of cerebrospinal fluid from rats (rCSF) after subarachnoid hemorrhage (SAH) on microcirculation of naïve rats. CSF from rats 24 hours after SAH (rCSF) was applied on the brain surface of naïve rats. Vascular diameters, including primary arterioles (pa), secondary arterioles (sa), and terminal arterioles (ta), were measured using a capillary videoscope at different time points following rCSF superfusion. (**A**,**B**) Marked vasoconstriction was observed in all types of arteriole at 1 min after rCSF application. (**C**) At 120 min, vasoconstriction persisted in the sa and ta. (**D**,**E**) The vasoconstrictive effect was alleviated by normal saline irrigation; i.e., the diameters of the sa and ta returned to the pre-rCSF level within 1 min of normal saline irrigation and persisted if normal irrigation continued. (a: primary arteriole, v- vein, arrow: secondary arteriole, arrow head: terminal arteriole).

To test the hypothesis that CSF from SAH rats could induce vasoconstriction, we applied CSF from SAH rats (rCSF) to the brain surface of a naïve rat and examined the brain surface microcirculation. Vasoconstriction developed after superfusion of rCSF, in contrast with rCSF from sham rats (Fig. 6A–C). The diameters of vessels decreased rapidly after rCSF superfusion, including the pa, sa, and ta. This pattern of vasoconstriction lasted for 30 min after rCSF superfusion (Fig. 6G). The affected vessels were dilated immediately after normal saline irrigation, but the vessels became constricted again 5 min later (Fig. 6D,E). The arterioles remained dilated during a period of continuous normal saline irrigation for 30 min (Fig. 6F). Rebound vasoconstriction became evident immediately after re-challenge with CSF from SAH rats.

**Figure 7:**
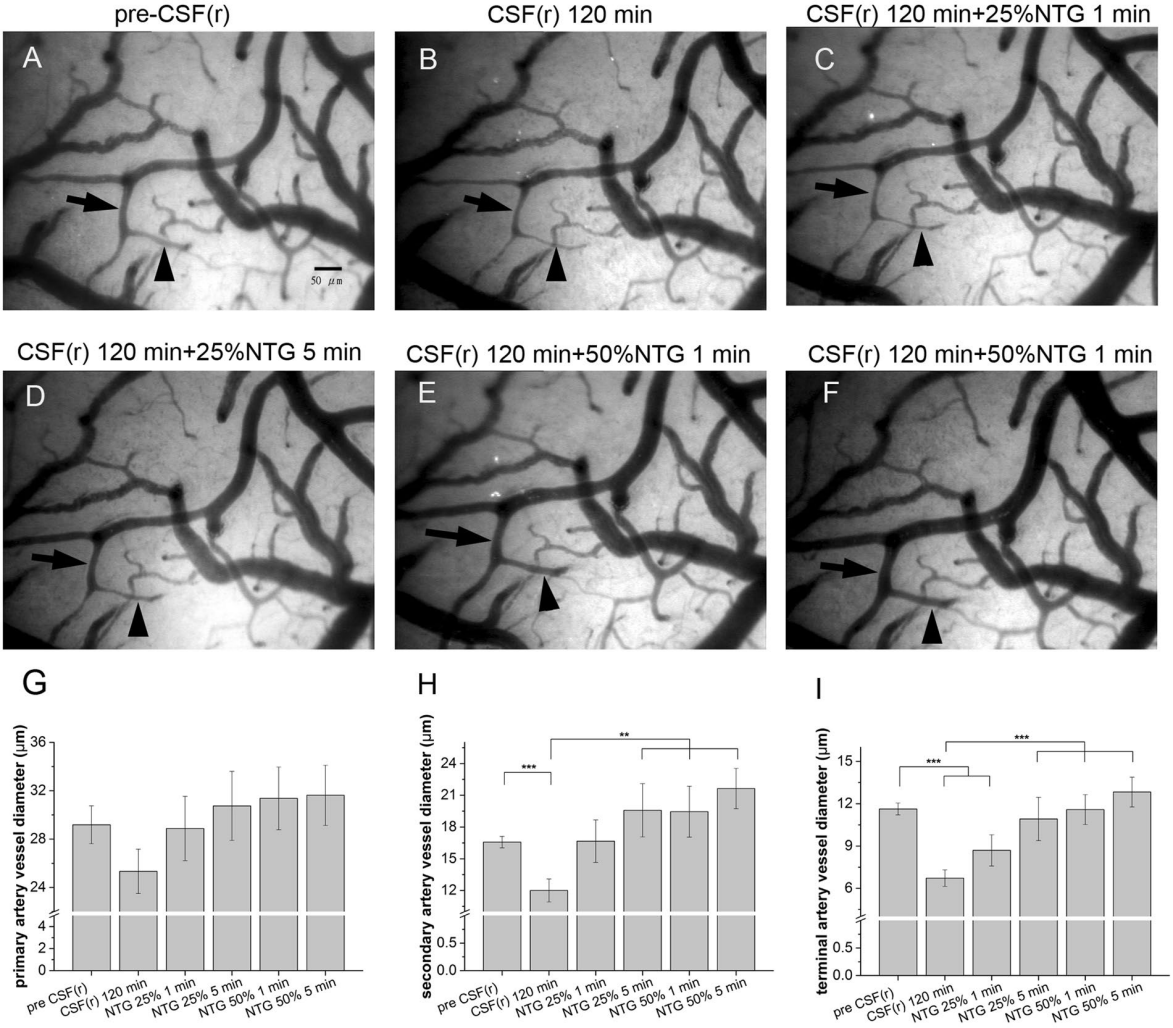
Effect of nitroglycerin (NTG) on the vasoconstrictive effect of cerebrospinal fluid (CSF) from rats (rCSF) with subarachnoid hemorrhage (SAH). Arteriolar diameters were measured by capillary videoscopy. rCSF of SAH rats was applied on the brain surface for 120 min (**B**) then NTG of different concentrations was added (**C**–**F**). The vasoconstrictive effect of rCSF was reversible by application of nitroglycerin in a dose-dependent manner. The vasodilation effects were particularly obvious in the secondary and terminal arterioles (**G**–**I**).

To examine the reversibility of vasoconstriction after CSF superfusion, nitroglycerin was applied locally to the rat brain surface. Figure 7A to F demonstrated (1) vasoconstriction of arterioles after rCSF superfusion, and (2) a dose-dependent pattern of vasodilation in both the sa and ta following local application of nitroglycerin. The diameters before and after administration of 25% nitroglycerin were 12.0 ± 1.1 vs. 19.6 ± 2.5 μm, *p* = 0.002 for the sa and 6.72 ± 0.6 vs. 10.9 ± 1.5 μm, *p* = 0.003 for the ta. In comparison with 25% nitroglycerin, the effect was more obvious on the ta with 50% nitroglycerin administration (12.8 ± 1.1 vs. 10.9 ± 1.5 μm, *p* = 0.023), but the degree of vasodilation remained the same in the sa (21.6 ± 1.9 vs. 19.5 ± 2.5 μm, *p* = 0.12) (Fig. 7G–I). These observations provided evidence of vasoconstrictive effects of rCSF of SAH rats on brain surface microcirculation.

### Superfusion of CSF from SAH patients on the brain surface of control rats induced similar microcirculatory changes: clinical significance of microcirculation in DIND

To understand the clinical significance of the effects of CSF from human subjects (hCSF) on cortical surface microcirculation, we performed similar experiments to those described above in the rat model, and analyzed the clinical profiles of the SAH patients. In total, 37 SAH patients were enrolled in the study. Table 1 lists the basic profiles of the study subjects, 14 of whom (37.8%) suffered from DIND during the course of SAH. Higher incidences of DIND (*p* < 0.001) and VP shunt insertion (*p* = 0.003) were observed in the patients with a poor functional outcome in comparison to those with a good functional outcome.

**Table 1:** Clinical features of patients after aneurysmal subarachnoid hemorrhage (SAH).

Outcome	Good (GOS 4–5) *n* = 26	Poor (GOS 1–3) *n* = 11	*p* value
Age (years)	57.5 ± 11.2	63.7 ± 8.8	0.113^≠2^
Males	5 (19.2%)	2(18.2%)	1.00^≠1^
IVH	19 (73.1%)	10 (90.9%)	0.391^≠1^
Acute hydrocephalus	15 (57.7%)	8 (72.7%)	0.477^≠1^
WFNS grade IV–V	11 (42.3%)	6 (54.5%)	0.719^≠1^
Posterior circulation	4(15.4%)	1 (9.1%)	1.00^≠1^
Coil/clipping	22/4	7/4	0.203^≠1^
Angiographic vasospasm	4(15.4%)	5 (45.5%)	0.066^≠1^
VP shunt	13 (50%)	11 (100%)	0.003^≠1^
DIND	5 (19.2%)	9 (91.8%)	0.001^≠1^

Good outcome: GOS 4–5; poor outcome: GOS 1–3.

^≠1^Fisher’s exact test, ^≠2^Two-sample *t* test. IVH: intraventricular hemorrhage; WFNS: World Federation of Neurological Surgeons SAH Grading; DIND: delayed ischemic neurological deficit.

**Figure 8:**
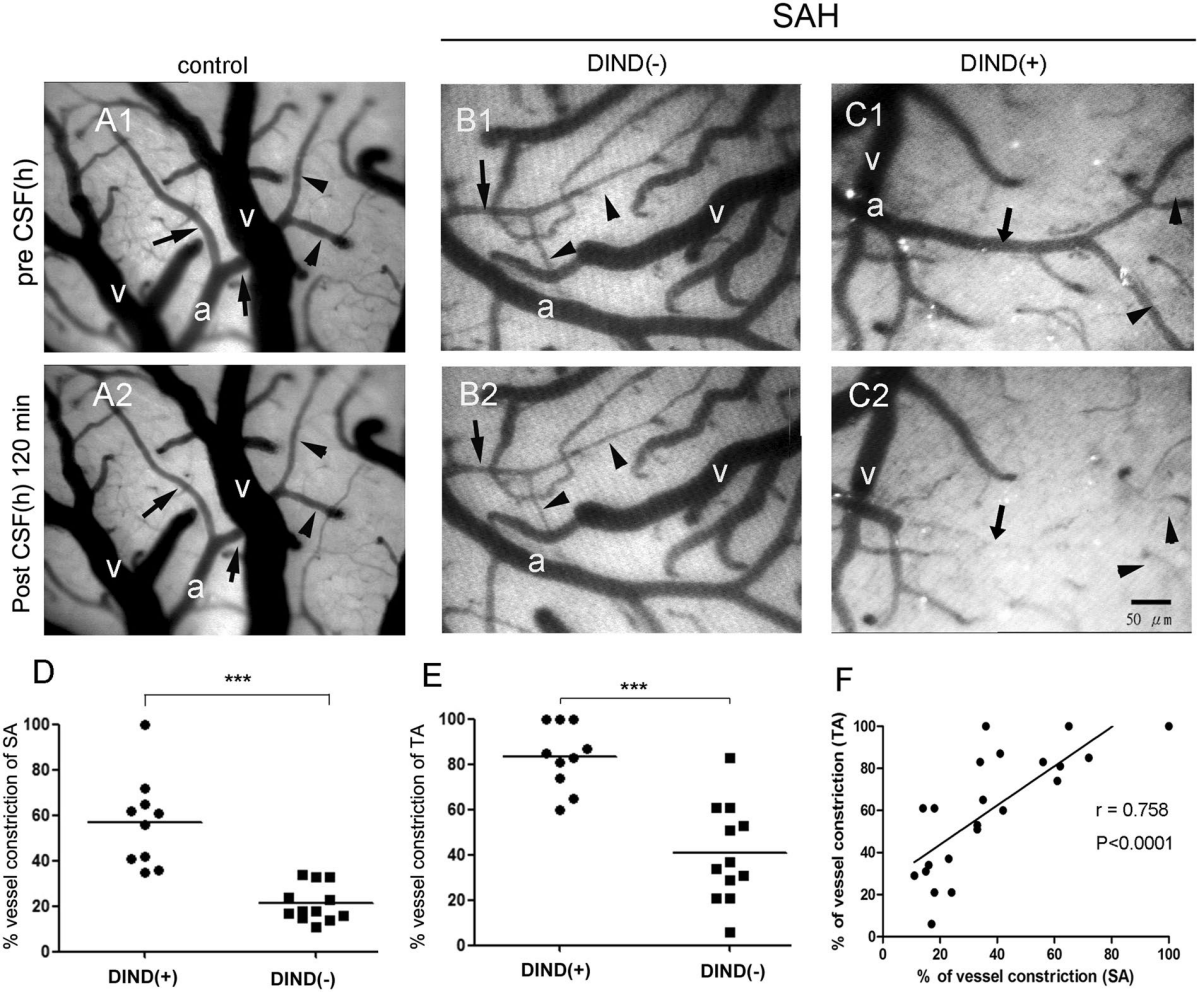
Effects of cerebrospinal fluid from human subjects (hCSF) with subarachnoid hemorrhage (SAH) on microcirculation of normal rats. This figure demonstrates microcirculation changes following superfusion of hCSF from SAH patients. Superfusion of hCSF from the control group resulted in no microcirculation changes (**A1**,**A2**), while hCSF from SAH patients without DIND (**B1**,**B2**) typically caused mild microcirculation changes of secondary arterioles (sa, arrow) and terminal arterioles (ta, arrowhead). After superfusion with hCSF from patients with DIND, constriction of the sa (arrow) and ta (arrowhead) occurred rapidly within 5 min. Such vasoconstriction of the sa and ta progressed to total occlusion 2 hours later (**C1**,**C2**). Statistical analysis showed that the degrees of vasoconstriction of the sa and ta were higher in the SAH patients with DIND than in those without DIND (**D**,**E**). The degrees of constriction of the sa and ta were highly correlated (**F**). (a: primary arteriole, v- vein, arrows: secondary arterioles, arrowheads: terminal arterioles).

To investigate whether the findings related to rCSF from SAH rats could be extended to human SAH, we further examined the effects of hCSF from SAH patients on the brain cortical microcirculation in naïve rats. hCSF from SAH patients induced vasoconstriction of the rat brain cortical microcirculation after hCSF superfusion for 2 hours (Fig. 6B,C) (Video) including the sa (37.5 ± 23.1%, range: 23 to 100%) and the ta (60.1 ± 28.4%, range: 35 to 100%). hCSF from controls and artificial CSF did not induce any significant changes in microcirculation (Fig. 8A). Furthermore, there was a significant correlation between sa and ta diameter (*r* = 0.758, *p* < 0.0001), indicating that both the sa and ta were affected (Fig. 8F). We next investigated the relationship between DIND and the effect of hCSF on microcirculatory changes. A significantly higher proportion of patients with DIND had vasoconstriction than patients without DIND in both the sa (57.0% ± 20.0% vs. 21.3% ± 8.1%, *p* < 0.001) and the ta (83.5% ± 14.23 vs. 40.8 ± 21.6%, *p* < 0.01) (Fig. 8D,E).

**Figure 9:**
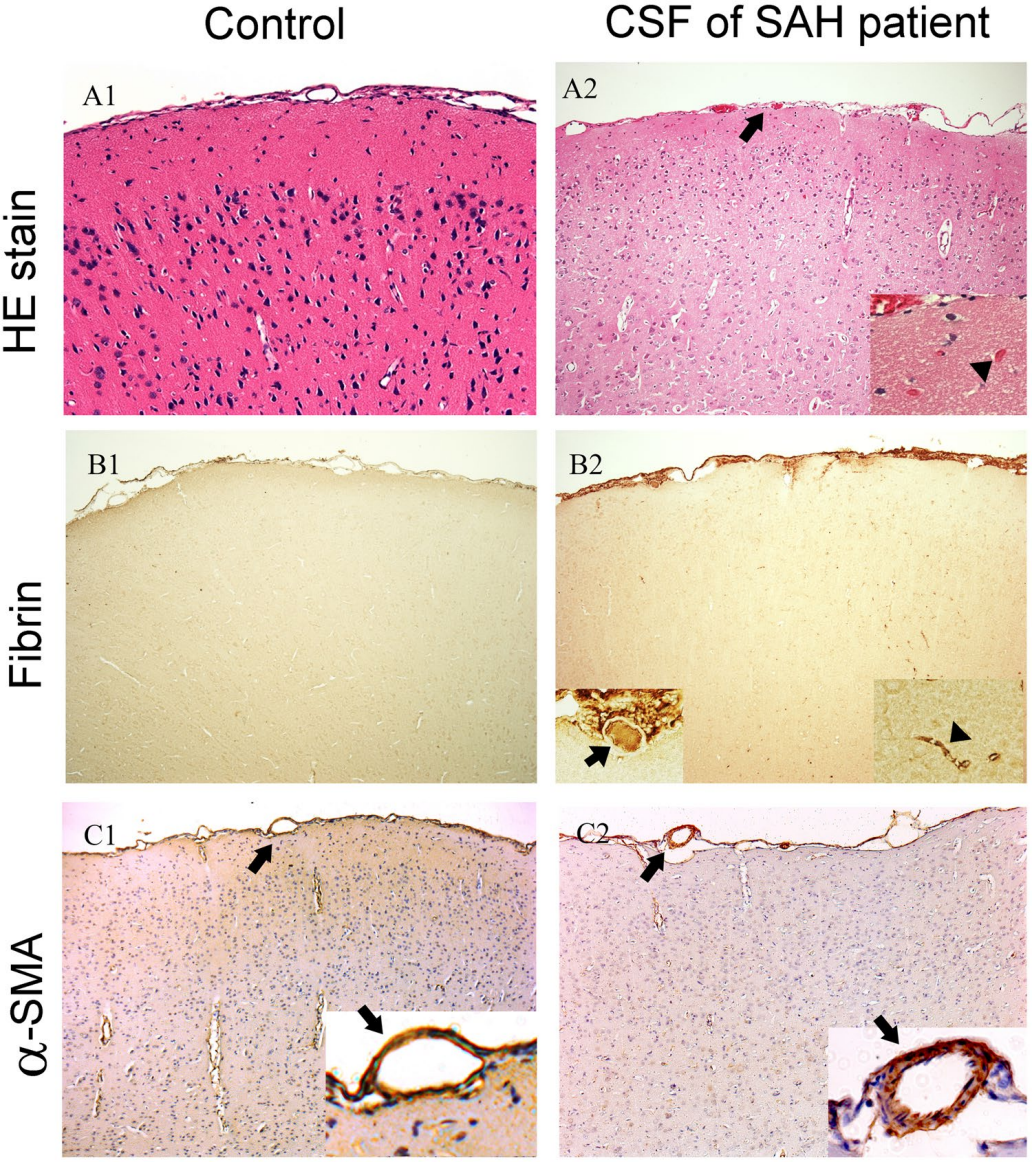
Pathological examination of rat brains 24 hours after superfusion of cerebrospinal fluid from human patients (hCSF) with subarachnoid hemorrhage (SAH). Rat brains were dissected and processed for paraffin-embedding. Sections were then stained with H&E and subjected to immunohistochemical analysis. (**A**) Pathological examination showed that hCSF from SAH patients induced thrombosis on the affected rat brain. Thrombus formation within the affected subcortical area (arrowhead) and cortical vessels (black arrow) was demonstrated by H&E staining. (**B**) Positive staining of fibrin confirmed the formation of thrombi inside the vessels of the cortical (black arrow) and subcortical (arrowhead) regions. (**C**) Vasoconstriction of the cortical arterioles (black arrow) was clearly observed by immunohistochemical staining of α-smooth muscle actin.

The structural correlates of the above observations were further substantiated by examining the pathology of the rat brain after application of hCSF from SAH patients on the brain surface. Vasoconstriction of cortical arterioles was clearly observed by immunofluorescence staining of α-SMA, as demonstrated in the rat SAH model (Fig. 9). In addition, application of hCSF from SAH patients on the rat brain surface resulted in platelet aggregation and acute thrombus formation within the affected parenchyma and vessels, as observed by H&E staining (Fig. 9). Positive staining of fibrin confirmed thrombus formation inside the vessels of the cortical and subcortical regions (Fig. 9).

## Discussion

This study investigated the effect of SAH on brain microcirculation and its clinical significance by establishing a platform to examine brain surface microcirculation. Specifically, we observed that (1) brain surface microcirculation was impaired in an animal model of SAH, (2) CSF from SAH rats was responsible for impaired brain surface microcirculation in naïve rats, and (3) CSF from SAH patients induced vasoconstriction of brain surface vasculatures, the degree of which was correlated with the development of DIND after SAH.

### Impaired brain surface microcirculation in a rat SAH model

In the rat SAH model created by cisternal injection of autologous blood, we made two important observations of SAH-induced impairment of brain surface microcirculation: (1) the most superficial part of the cerebral cortex was affected most by SAH; and (2) the smallest arterioles were most vulnerable and tended to constrict after SAH.

The surface of the cerebral cortex is enriched with blood vessels forming arteriole anastomoses^25^. Furthermore, cortical arteries penetrate into the cerebral cortex at right angles, and then branch into capillary networks^29^. Each of these surface networks is highly interconnected through anastomoses among pial arterioles^30^. The capillary networks in the gray matter of the cerebral cortex are denser than those in the white matter^31^. Similar to the vascular supply on the surface of the human cerebral cortex, this study showed that in rats, abundant collaterals between terminal arterioles and secondary arterioles on the rat brain surface formed a foundation for the detrimental effects of CSF from SAH rats and patients. Once the vasoconstriction of secondary and terminal arterioles becomes profound and diffuse, the communicating networks of capillaries might fail to provide adequate perfusion, leading to ischemia. The vascular supply by major cerebral arteries in humans with a diameter of 100–200 μm plays a key role in the cerebral circulation, including vascular resistance, and is regulated by neuronal factors^32,33^. Chemical mediators regulate arterioles of 50 μm or less in diameter. These findings supported the hypothesis that microcirculation impairment occurs after SAH, leading to hypoperfusion in the capillary networks underneath.

Several studies have provided circumferential evidence indicating vasoconstriction in small arterioles in SAH patients^34,35^ and animal studies^1,3,36^. However, the importance of microcirculation alteration after SAH has not been explored in detail. For example, a significant discrepancy between angiographic vasospasm of major cerebral arteries and outcome after SAH was observed in the CONSIOUS-1 trial^37^. These observations suggested that vasospasm was not the major pathognomonic factor of the detrimental outcome of SAH. Prior animal studies have documented the existence of clots in small vessels following SAH^38^. Several autopsy studies have demonstrated that microclot density is highly correlated with the severity of infarction^39,40^. In 70% of surveyed patients, microthrombotic signals could be detected by transcranial Doppler studies^41^. Wang *et al*. further demonstrated that simvastatin administration attenuates cerebral vasospasm and alleviates microthrombosis in the late phase of SAH^38^. Friedrich *et al*. indicated that approximately 30% of constricted arterioles were occluded by microthrombi, and that the smaller the arteriole, the more pronounced the vasoconstriction^36^. Although causation has yet to be determined, the presence of thromboembolism after SAH represents a potential target for therapeutic intervention. These findings also explain the poor clinical improvement despite vasospasm of major cerebral arteries being attenuated by angiography after angioplasty or endothelin antagonist treatment.

### Vasoconstrictive effects of CSF from SAH rats and patients

We demonstrated that the effect of application of CSF from SAH rats on naïve rats reproduced the same microcirculation changes in the rat SAH model (Supplementary video). Furthermore, this vasoconstriction was reversible by normal saline flushing. These findings supported the hypothesis that the CSF content after SAH contributes to impairment of microcirculation. In this study, we demonstrated diffuse thrombosis of the microcirculation underneath the area of CSF superfusion. The results indicated that CSF from SAH rats and patients had a fast-acting effect on microcirculation. Furthermore, the impact was not only existent on the surface of the cortex, but also extended to arterioles and capillaries of the deep cerebral cortex; for example, the regional cerebral blood flow and the partial oxygen pressure of the brain tissue were significantly decreased at 2–5 mm underneath the cerebral cortex. All these findings suggested that CSF from SAH rats and patients caused direct impairment of cortical surface microcirculation.

Once the microcirculation had been disrupted, the arterioles were refractory to normal saline irrigation in the animal model of SAH, and exhibited rebound constriction if CSF challenge was resumed. CSF from SAH patients induced rapid vasoconstriction of brain surface vasculatures, which could only be reversed after extensive irrigation with normal saline and nitroglycerin. Despite the fact that the microcirculation changes were reversible, persistent vasoconstriction of arterioles could become refractory, and may even cause diffuse thrombosis. This observation carries clinical implications: if the chemical mediators of arteriolar constriction in the CSF of SAH rats and patients could be removed, SAH-induced ischemic lesions could be alleviated, and the prognosis of SAH patients could be improved.

### Correlation of CSF from SAH patients with DIND

This study was the first to ascertain that vasoconstriction of secondary and terminal arterioles was highly correlated with DIND in SAH patients. Substances in the CSF have been proposed to be responsible for DIND by causing vasospasm of major cerebral arteries^42^. Additional mechanisms include progressive ischemic damage associated with cortical spreading depolarizations^43–45^, an increase in the endothelin 1 level, a decrease in the NO content^46^, disruption of the blood–brain barrier, activation of pathways related to apoptosis and inflammation^47–49^ and thromboembolism^36,38,50,51^.

The vasoconstrictive effect of CSF on the 3rd day after SAH in this study was predictive of DIND onset at day 5 after SAH. This finding suggested that the causitive agent of DIND appeared within 3 days after SAH^11,13^, and implied that intervention should be initiated before the onset of DIND. These findings may underlie the clinical observations that once DIND has occurred, few patients exhibit improvement within a short time frame. Pennings *et al*. showed that microcirculation in patients with SAH had unpredictable response patterns to papaverin^35^, and in some cases, “rebound” vasoconstriction even occurred, suggesting increased contractility of the microcirculation. These results may indicate a new direction for the treatment of DIND after SAH.

In this study, only six of the 12 patients with DIND had angiographic vasospasm. Intra-arterial Nimotopp was routinely administered if angiography proved vasospasm in symptomatic patients, but the improvement was limited. Though symptomatic vasospasm of major cerebral arteries is traditionally considered to be the major cause of DIND, there is a growing body of reports indicating discrepancies between DIND and major artery vasospasm. Hanggi *et al*. also demonstrated an improved clinical outcome after continuous lumboventricular lavage, but there was no obvious effect on the rate of angiographic vasospasm^52^. In this study, the impairment of microcirculation after superfusion of CSF from SAH patients was highly correlated with DIND. These findings indicated pathologic and physiologic mechanisms in patients suffering DIND without angiographic vasospasm. The development of DIND independent of angiographic vasospasm further substantiated the important role of impaired microcirculation on the cortical surface after SAH.

### Study limitation

There were several limitations in this study. First, ICP may have a rapid change after craniotomy and durotomy in our SAH animal model. To minimize its effect, we made the size of craniotomy as small as possible. On the other hand, the step of craniotomy and durotomy in our SAH model has two advantages, the resolution of small cortical vessel is much better than that without craniotomy. The other one is that we can apply the medication on cortical vessels directly to test its effect on post SAH vessel constriction. Second, the part of our clinical study is an observational study with a small sample size. Further studies with prospective study design and larger sample sizes would be encouraged to validate our study result.

In summary, this study demonstrated impaired microcirculation on brain surface arterioles in a rat model of SAH. CSF from SAH rats and patients was responsible for impairment of brain surface microcirculation.The findings of this study provided a foundation for exploration of the molecular characteristics responsible for microcirculatory impairment induced by CSF after SAH.

## Electronic supplementary material

Effects of cerebrospinal fluid from human subjects (hCSF) with subarachnoid hemorrhage (SAH) on microcirculation of normal rats.

## References

[1] Naraoka M, Matsuda N, Shimamura N, Asano K, Ohkuma H (2014). The role of arterioles and the microcirculation in the development of vasospasm after aneurysmal SAH. BioMed research international.

[2] Sabri M (2012). Mechanisms of microthrombi formation after experimental subarachnoid hemorrhage. Neuroscience.

[3] Sun BL (2009). Dynamic alterations of cerebral pial microcirculation during experimental subarachnoid hemorrhage. Cellular and molecular neurobiology.

[4] Sakr, Y., Dubois, M. J., De Backer, D., Creteur, J. & Vincent, J. L. Persistent microcirculatory alterations are associated with organ failure and death in patients with septic shock. *Crit Care Med* **32**, 1825–1831, doi:00003246-200409000-00002 (2004). 10.1097/01.ccm.0000138558.16257.3f 15343008

[5] Ohkuma, H., Itoh, K., Shibata, S. & Suzuki, S. Morphological changes of intraparenchymal arterioles after experimental subarachnoid hemorrhage in dogs. *Neurosurgery* **41**, 230–235; discussion 235–236 (1997). 10.1097/00006123-199707000-00036 9218311

[6] Titova E, Ostrowski RP, Zhang JH, Tang J (2009). Experimental models of subarachnoid hemorrhage for studies of cerebral vasospasm. Neurol Res.

[7] Megyesi, J. F., Vollrath, B., Cook, D. A. & Findlay, J. M. *In vivo* animal models of cerebral vasospasm: a review. *Neurosurgery* **46**, 448–460; discussion 460–441 (2000). 10690735

[8] Saveland H (1992). Overall outcome in aneurysmal subarachnoid hemorrhage. A prospective study from neurosurgical units in Sweden during a 1-year period. J Neurosurg.

[9] Germanson, T. P., Lanzino, G., Kongable, G. L., Torner, J. C. & Kassell, N. F. Risk classification after aneurysmal subarachnoid hemorrhage. *Surg Neurol* **49**, 155–163, doi:S0090-3019(97)00337-6 (1998). 10.1016/s0090-3019(97)00337-6 9457265

[10] Kassell NF (1990). The International Cooperative Study on the Timing of Aneurysm Surgery. Part 1: Overall management results. J Neurosurg.

[11] Wang KC (2015). Intrathecal lactate predicting hydrocephalus after aneurysmal subarachnoid hemorrhage. The Journal of surgical research.

[12] Suarez JI, Tarr RW, Selman WR (2006). Aneurysmal subarachnoid hemorrhage. N Engl J Med.

[13] Wang KC (2014). Prognostic value of intrathecal heme oxygenase-1 concentration in patients with Fisher Grade III aneurysmal subarachnoid hemorrhage. J Neurosurg.

[14] Fisher CM, Roberson GH, Ojemann RG (1977). Cerebral vasospasm with ruptured saccular aneurysm–the clinical manifestations. Neurosurgery.

[15] Harders AG, Gilsbach JM (1987). Time course of blood velocity changes related to vasospasm in the circle of Willis measured by transcranial Doppler ultrasound. J Neurosurg.

[16] Okada Y (1994). Evaluation of angiographic delayed vasospasm due to ruptured aneurysm in comparison with cerebral circulation time measured by IA-DSA. No Shinkei Geka.

[17] van Gijn J, Kerr RS, Rinkel GJ (2007). Subarachnoid haemorrhage. Lancet.

[18] Fergusen, S. & Macdonald, R. L. Predictors of cerebral infarction in patients with aneurysmal subarachnoid hemorrhage. *Neurosurgery* **60**, 658–667; discussion 667, 10.1227/01.NEU.0000255396.23280.3100006123-200704000-00011 (2007). 10.1227/01.NEU.0000255396.23280.31 17415202

[19] Rabinstein AA (2004). Predictors of cerebral infarction in aneurysmal subarachnoid hemorrhage. Stroke.

[20] Kivisaari RP (2001). MR imaging after aneurysmal subarachnoid hemorrhage and surgery: a long-term follow-up study. AJNR Am J Neuroradiol.

[21] Clyde, B. L., Resnick, D. K., Yonas, H., Smith, H. A. & Kaufmann, A. M. The relationship of blood velocity as measured by transcranial doppler ultrasonography to cerebral blood flow as determined by stable xenon computed tomographic studies after aneurysmal subarachnoid hemorrhage. *Neurosurgery* **38**, 896–904; discussion 904–895 (1996). 10.1097/00006123-199605000-00008 8727814

[22] Lysakowski C, Walder B, Costanza MC, Tramer MR (2001). Transcranial Doppler versus angiography in patients with vasospasm due to a ruptured cerebral aneurysm: A systematic review. Stroke.

[23] Woitzik J (2012). Delayed cerebral ischemia and spreading depolarization in absence of angiographic vasospasm after subarachnoid hemorrhage. J Cereb Blood Flow Metab.

[24] Solomon RA, Antunes JL, Chen RY, Bland L, Chien S (1985). Decrease in cerebral blood flow in rats after experimental subarachnoid hemorrhage: a new animal model. Stroke.

[25] Mascia L (2003). The accuracy of transcranial Doppler to detect vasospasm in patients with aneurysmal subarachnoid hemorrhage. Intensive Care Med.

[26] Nonaka H (2003). Microvasculature of the human cerebral meninges. Neuropathology: official journal of the Japanese Society of Neuropathology.

[27] Maloney-Wilensky E, Le Roux P (2010). The physiology behind direct brain oxygen monitors and practical aspects of their use. Childs Nerv Syst.

[28] Vergouwen MD (2010). Definition of delayed cerebral ischemia after aneurysmal subarachnoid hemorrhage as an outcome event in clinical trials and observational studies: proposal of a multidisciplinary research group. Stroke.

[29] Reina-De La Torre F, Rodriguez-Baeza A, Sahuquillo-Barris J (1998). Morphological characteristics and distribution pattern of the arterial vessels in human cerebral cortex: a scanning electron microscope study. The Anatomical record.

[30] Carrera, E. *et al*. Transcranial Doppler for predicting delayed cerebral ischemia after subarachnoid hemorrhage. *Neurosurgery* **65**, 316–323; discussion 323–314, 10.1227/01.NEU.0000349209.69973.8800006123-200908000-00013 (2009). 10.1227/01.NEU.0000349209.69973.88 19625911

[31] Groner W (1999). Orthogonal polarization spectral imaging: a new method for study of the microcirculation. Nat Med.

[32] Gulbenkian S, Uddman R, Edvinsson L (2001). Neuronal messengers in the human cerebral circulation. Peptides.

[33] Sandor P (1999). Nervous control of the cerebrovascular system: doubts and facts. Neurochemistry international.

[34] Pennings FA, Bouma GJ, Ince C (2004). Direct observation of the human cerebral microcirculation during aneurysm surgery reveals increased arteriolar contractility. Stroke.

[35] Pennings FA, Albrecht KW, Muizelaar JP, Schuurman PR, Bouma GJ (2009). Abnormal responses of the human cerebral microcirculation to papaverin during aneurysm surgery. Stroke.

[36] Friedrich B, Muller F, Feiler S, Scholler K, Plesnila N (2012). Experimental subarachnoid hemorrhage causes early and long-lasting microarterial constriction and microthrombosis: an *in-vivo* microscopy study. J Cereb Blood Flow Metab.

[37] Macdonald RL (2008). Clazosentan to overcome neurological ischemia and infarction occurring after subarachnoid hemorrhage (CONSCIOUS-1): randomized, double-blind, placebo-controlled phase 2 dose-finding trial. Stroke.

[38] Wang, Z. *et al*. Influence of simvastatin on microthrombosis in the brain after subarachnoid hemorrhage in rats: a preliminary study. *Ann Clin Lab Sci* **40**, 32–42, doi:40/1/32 (2010). 20124328

[39] Stein, S. C., Browne, K. D., Chen, X. H., Smith, D. H. & Graham, D. I. Thromboembolism and delayed cerebral ischemia after subarachnoid hemorrhage: an autopsy study. *Neurosurgery* **59**, 781–787; discussion 787–788, 10.1227/01.NEU.0000227519.27569.45 (2006). 10.1227/01.NEU.0000227519.27569.45 16915120

[40] Suzuki, S. *et al*. Cerebral microthrombosis in symptomatic cerebral vasospasm–a quantitative histological study in autopsy cases. *Neurol Med Chir* (*Tokyo*) **30**, 309–316, doi:JST.Journalarchive/nmc1959/30.309 (1990). 10.2176/nmc.30.309 1699146

[41] Romano JG (2008). Microemboli in aneurysmal subarachnoid hemorrhage. J Neuroimaging.

[42] Ohkuma H, Manabe H, Tanaka M, Suzuki S (2000). Impact of cerebral microcirculatory changes on cerebral blood flow during cerebral vasospasm after aneurysmal subarachnoid hemorrhage. Stroke.

[43] Dreier JP (1998). Nitric oxide scavenging by hemoglobin or nitric oxide synthase inhibition by N-nitro-L-arginine induces cortical spreading ischemia when K+ is increased in the subarachnoid space. J Cereb Blood Flow Metab.

[44] Dreier JP (2000). Products of hemolysis in the subarachnoid space inducing spreading ischemia in the cortex and focal necrosis in rats: a model for delayed ischemic neurological deficits after subarachnoid hemorrhage?. J Neurosurg.

[45] Dreier JP (2006). Delayed ischaemic neurological deficits after subarachnoid haemorrhage are associated with clusters of spreading depolarizations. Brain.

[46] Pluta RM (2005). Delayed cerebral vasospasm and nitric oxide: review, new hypothesis, and proposed treatment. Pharmacol Ther.

[47] Gallia GL, Tamargo RJ (2006). Leukocyte-endothelial cell interactions in chronic vasospasm after subarachnoid hemorrhage. Neurol Res.

[48] Hashimoto T, Meng H, Young WL (2006). Intracranial aneurysms: links among inflammation, hemodynamics and vascular remodeling. Neurol Res.

[49] Wang, K. C. *et al*. Cerebrospinal fluid high mobility group box 1 is associated with neuronal death in subarachnoid hemorrhage. *J Cereb Blood Flow Metab*, 10.1177/0271678X16629484 (2016). 10.1177/0271678X16629484 PMC5381442 26823474

[50] Rabinstein AA, Weigand S, Atkinson JL, Wijdicks EF (2005). Patterns of cerebral infarction in aneurysmal subarachnoid hemorrhage. Stroke.

[51] Stein, S. C., Levine, J. M., Nagpal, S. & LeRoux, P. D. Vasospasm as the sole cause of cerebral ischemia: how strong is the evidence? *Neurosurg Focus* **21**, E2, doi:210302 (2006). 10.3171/foc.2006.21.3.2 17029341

[52] Hanggi D, Liersch J, Turowski B, Yong M, Steiger HJ (2008). The effect of lumboventricular lavage and simultaneous low-frequency head-motion therapy after severe subarachnoid hemorrhage: results of a single center prospective Phase II trial. J Neurosurg.

